# Higher afamin concentrations are associated with higher fatty liver indices: Population‐based KORA F4/FF4 study

**DOI:** 10.1111/eci.70095

**Published:** 2025-07-09

**Authors:** Corinna Niersmann, Anna Zhu, Haifa Maalmi, Xinting Cai, Jana Nano, Wolfgang Rathmann, Wolfgang Koenig, Toshinari Takamura, Barbara Kollerits, Hans Dieplinger, Annette Peters, Michael Roden, Florian Kronenberg, Barbara Thorand, Christian Herder

**Affiliations:** ^1^ Institute for Clinical Diabetology German Diabetes Center, Leibniz Center for Diabetes Research at Heinrich Heine University Düsseldorf Düsseldorf Germany; ^2^ German Center for Diabetes Research (DZD) München‐Neuherberg Germany; ^3^ Institute of Epidemiology Helmholtz Zentrum München, German Research Center for Environmental Health Neuherberg Germany; ^4^ Institute for Medical Information Processing, Biometry and Epidemiology – IBE, Faculty of Medicine Ludwig‐Maximilians University of Munich Munich Germany; ^5^ Institute for Biometrics and Epidemiology, German Diabetes Center Leibniz Center for Diabetes Research at Heinrich Heine University Düsseldorf Düsseldorf Germany; ^6^ German Center for Cardiovascular Disease Research (DZHK), Partner Site Munich Heart Alliance Munich Germany; ^7^ German Heart Center, Munich Technical University of Munich Munich Germany; ^8^ Institute of Epidemiology and Medical Biometry University of Ulm Ulm Germany; ^9^ Department of Endocrinology and Metabolism Kanazawa University, Graduate School of Medical Sciences Kanazawa Ishikawa Japan; ^10^ Institute of Genetic Epidemiology Medical University of Innsbruck Innsbruck Austria; ^11^ Department of Endocrinology and Diabetology, Medical Faculty and University Hospital Düsseldorf Heinrich Heine University Düsseldorf Düsseldorf Germany

**Keywords:** afamin, fatty liver indices, fibrosis, MASLD, SLD

## Abstract

**Background:**

Previous studies suggest that afamin is associated with steatotic liver diseases (SLD). However, the exact role of afamin in SLD development and fibrogenesis remains unclear. Potential modifying effects of sex and glucose tolerance status have also not been examined. Therefore, we investigated the associations of afamin with steatotic liver diseases and fibrosis defined by non‐invasive tests and assessed for possible effect modifications.

**Methods:**

This study included 3080 participants from the population‐based KORA F4/FF4 cohort. Cross‐sectional and prospective associations (follow‐up time 6.5 years) between afamin and NAFLD liver fat score (NAFLD LFS), hepatic steatosis index, fatty liver index and the fibrosis‐4 index were assessed using multiple linear regression models. Models were adjusted for age, sex, body mass index, smoking status, alcohol consumption, physical activity, metabolic parameters, medication and subclinical inflammation.

**Results:**

In the cross‐sectional analysis, afamin concentrations were positively associated with NAFLD LFS (*β* = .32; 95% CI .27–.37), hepatic steatosis index (*β* = .33; 95% CI .26–.39) and fatty liver index (*β* = 1.78; 95% CI 1.47–2.08) (all *p* < .001), but not with fibrosis‐4 index. In the prospective analysis, higher afamin levels were associated with a higher increase only in NAFLD LFS (*p* < .001). Cross‐sectional and prospective associations between afamin and NAFLD LFS were more pronounced in men than in women (*p*_interaction_ < .001 and .022; respectively). Cross‐sectional associations between afamin and NAFLD LFS were also stronger in individuals with prediabetes or diabetes compared to those with normal glucose tolerance (*p*_interaction_ < .001).

**Conclusion:**

Higher afamin concentrations are positively associated with NAFLD LFS with potential effect modification by sex and glucose tolerance status.

## Graphical abstract

The present study aimed to investigate the associations between afamin and fatty liver and fibrosis indices and to study potential modifying effects of sex and glucose tolerance status on these associations in individuals from the population‐based KORA F4/FF4 cohort. Higher afamin concentrations were positively associated with NAFLD LFS in both cross‐sectional and prospective analyses, with potential effect modification by sex and glucose tolerance status. These findings suggest that afamin may be a novel biomarker for the non‐invasive diagnosis of MASLD.

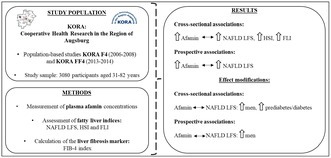

## 1 INTRODUCTION

Metabolic dysfunction‐associated steatotic liver disease (MASLD), formerly known as non‐alcoholic fatty liver disease (NAFLD), is the most common liver disease worldwide and it affects more than a quarter of the global population.^1^, ^2^ An almost complete overlap between NAFLD and MASLD was found using a NAFLD database and data from the National Health and Nutrition Examination Survey, suggesting that NAFLD and MASLD terminologies can be used interchangeably.^3^ Metabolic dysfunction‐associated steatohepatitis (MASH), formerly known as non‐alcoholic steatohepatitis (NASH), represents the more severe form of MASLD and is associated with an increased risk of liver fibrosis and cirrhosis.^4^, ^5^

Dysregulation of hepatokines such as fetuin‐A, fetuin‐B, fibroblast growth factor 21, selenoprotein P or follistatin has been observed in people with MASLD.^6^, ^7^ However, there are inconsistent or limited data on the associations between some of these hepatokines and MASLD.^8^, ^9^ None of these hepatokines are currently used as a reliable MASLD marker in clinical practice. Thus, there is an unmet clinical need for the identification of new hepatokines that are dysregulated in humans with MASLD and could serve as potential biomarkers for prevalent and incident MASLD. Afamin, discovered as the fourth member of the human albumin gene family with potential vitamin E‐binding and ‐transporting properties, could represent a promising candidate. This human plasma glycoprotein is mainly expressed in the liver and secreted into the circulation^10^ but it can also be found in extravascular fluids.^11^

Human population‐based studies reported positive associations between afamin concentrations and insulin resistance (IR) as well as prevalence and incidence of both type 2 diabetes (T2D) and the metabolic syndrome (MetS).^12^, ^13^ These data are strengthened by an animal study showing that transgenic mice overexpressing the human afamin gene have higher body weight and higher circulating lipid and glucose concentrations.^13^

Recently, afamin has already been suggested as a potential marker for MASLD.^14^, ^15^ However, the current studies are either based on relatively small sample sizes or do not include older adults. Moreover, MASLD has only been diagnosed using abdominal ultrasound examination in these available studies, although ultrasound‐based liver fat quantification provides rather semi‐quantitative estimates.^16^ Furthermore, MASLD is more prevalent in men than in women^17^, ^18^ and there is an increased MASLD prevalence in people with T2D^19^, ^20^ and potential modifying effects of sex and glucose tolerance status have also not been examined in the context of afamin and MASLD. Finally, despite the increased risk for developing liver fibrosis in individuals with MASLD,^2^ no previous studies examined the relevance of afamin for fibrogenesis. Therefore, the knowledge on afamin's role in MASLD and liver fibrosis is currently not well understood. Further data from large‐scale prospective studies considering sex and glucose tolerance status as possible effect modifiers in older individuals and using other MASLD screening tools are required.

We hypothesised that higher plasma afamin concentrations are associated with fatty liver and fibrosis indices and that sex and glucose tolerance status act as effect modifiers. In detail, this study aimed (i) to assess the cross‐sectional associations between plasma afamin and NAFLD liver fat score (NAFLD LFS), hepatic steatosis index (HSI), fatty liver index (FLI) and fibrosis‐4 (FIB‐4) index, (ii) to investigate if higher afamin levels are associated with higher increases in these indices during 6.5 years of follow‐up and (iii) to study possible modifying effects of sex and glucose tolerance status on these associations in individuals from the population‐based KORA F4/FF4 cohort.

## 2 METHODS

### 2.1 Study population and study design

The investigations of this study are based on data from the Cooperative Health Research in the Region of Augsburg (KORA) F4 (2006–2008) and KORA FF4 (2013–2014) cohort study. Both studies represent follow‐up examinations of the population‐based KORA S4 study (1999–2001) and were conducted in the city of Augsburg (Germany) and two surrounding countries. The design of both KORA surveys has been described in detail before.^21^, ^22^ All investigations were performed in accordance with the Declaration of Helsinki, including written informed consent from all participants. The study was approved by the ethics committee of the Bavarian Chamber of Physicians (Munich, Germany).

**FIGURE 1:**
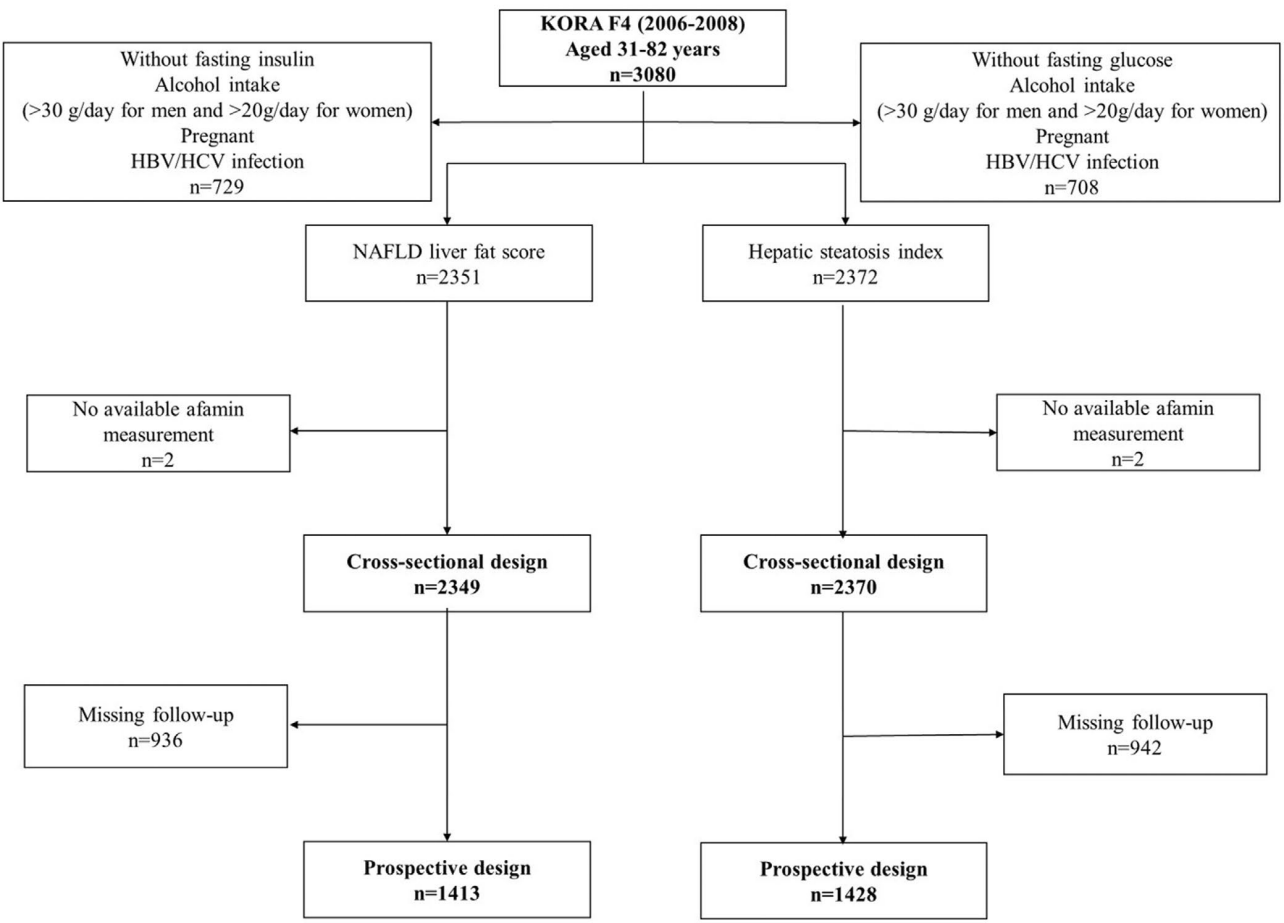
Flow chart of the inclusion process of the study participants for the cross‐sectional and prospective analyses of NAFLD liver fat score and hepatic steatosis index. Descriptions of the study design and populations for the analyses of FLI and FIB‐4 index are shown in Figures S1 and S2, respectively. HBV, hepatitis B virus; HCV, hepatitis C virus; KORA, Cooperative Health Research in the Region of Augsburg; NAFLD, non‐alcoholic fatty liver disease.

The selection of the study population is illustrated in Figure 1, Figures S1 and S2. Briefly, the KORA F4 study included 3080 participants aged 31–82 years. The exclusion of participants (i) with non‐fasting blood samples, (ii) with alcohol intake (>30 g/day for men and >20 g/day for women), (iii) with an existing pregnancy, (iv) with hepatitis B virus or hepatitis C virus infections, (v) with missing data for NAFLD LFS, HSI, FLI or FIB‐4 index, or (vi) with missing afamin concentrations resulted in sample sizes of 2349 or 2370 individuals for the cross‐sectional analysis of NAFLD LFS or HSI, respectively. After consideration of all the above‐mentioned exclusion criteria except alcohol intake, a total of 2999 or 3016 individuals remained for the cross‐sectional analysis of FLI or FIB‐4 index, respectively. Of those, participants with non‐participation in KORA FF4 were further excluded, leaving 1413, 1428, 2092 or 2111 individuals for the prospective analysis of NAFLD LFS, HSI, FLI or FIB‐4 index, respectively.

### 2.2 Assessment of fatty liver and fibrosis indices

To assess the risk of SLD, the indices NAFLD LFS, HSI and FLI were assessed as non‐invasive tools. NAFLD LFS was determined using an equation that includes the presence of the MetS, T2D, fasting serum insulin, fasting serum aspartate transaminase (AST) and the AST/alanine transaminase (ALT) ratio. A score greater than −.64 predicted NAFLD with a sensitivity of 86% and a specificity of 71%.^23^

The HSI was calculated as described^23^ based on the ALT‐to‐AST ratio, body mass index (BMI), sex and diabetes status. Values >36 detected NAFLD with a specificity of 92.4%, and values <30 ruled out NAFLD with a sensitivity of 93.1%.^24^

FLI was considered a secondary outcome and calculated using an algorithm based on BMI, waist circumference, triglycerides and gamma‐glutamyl‐transferase (GGT). An FLI <30 rules out hepatic steatosis and an FLI ≥60 rules in hepatic steatosis.^25^

Lastly, the FIB‐4 index was determined to distinguish between the presence and absence of advanced fibrosis. The calculation of the FIB‐4 index is based on age, ALT, AST and platelet count. A cut‐off value of the FIB‐4 index <1.30 excludes and FIB‐4 values >2.67 predict the presence of advanced fibrosis.^26^

### 2.3 Measurement of plasma afamin concentrations

Fasting plasma samples collected at KORA F4 were used to measure afamin concentrations centrally in the laboratory at the Medical University of Innsbruck (Austria) using custom‐made double‐antibody sandwich enzyme‐linked immunosorbent assays as described in detail elsewhere.^13^ Intra‐ and inter‐assay coefficients of variation were 3.3% and 6.2%, respectively.^13^

### 2.4 Assessment of covariates

The assessment of anthropometric, metabolic and lifestyle factors has been reported previously.^21^, ^22^ All covariates were determined at the baseline visit (F4). A more detailed description of the covariate assessment can be found in Appendix S1.

### 2.5 Statistical analysis

Baseline characteristics of the participants are given as mean ± standard deviation for continuous variables or counts (percentage) for categorical variables. *p*‐values for differences between men and women were obtained using Wilcoxon rank‐sum tests for continuous variables and chi‐square tests for categorical variables.

The cross‐sectional associations between afamin concentrations and all liver indices were assessed using multivariable linear regression analyses. As indicated in the table captions, four different adjustment models were built. Model 1 represents the unadjusted model. Covariates for models 2 and 3 included age, sex, smoking status, alcohol consumption, physical activity, BMI, total cholesterol, hypertension, triglycerides, total cholesterol to high‐density lipoprotein (HDL) ratio, estimated glomerular filtration rate (eGFR), lipid‐lowering medication, diabetes status and regular use of non‐steroidal anti‐inflammatory drugs (NSAIDs) as potential confounders with the following adaptations. Adjustment in model 2 focused on all the above‐mentioned covariates except those included in the equations to calculate the respective liver indices and except total cholesterol for the HSI, FLI and FIB‐4 index. The main model 3 was adjusted for all covariates except for total cholesterol, but additionally for the covariates used to calculate the respective indices. Thus, for the NAFLD LFS model 3 was additionally adjusted for hypertension, triglycerides, total cholesterol to HDL ratio and diabetes. For the HSI model 3 was additionally adjusted for sex, BMI and diabetes. For the FLI model 3 was additionally adjusted for BMI and triglycerides. For the FIB‐4 index, model 3 was additionally adjusted for age. For all four indices, the extended model 4 was based on model 3 with additional adjustment for high‐sensitivity C‐reactive protein (hsCRP) and interleukin (IL)‐18. The rationale of the extended model was to provide a model that contained additional factors associated with the risk of SLD and fibrosis. However, we cannot exclude that the effect estimates from model 4 were over‐adjusted.

Afamin concentrations and all covariates used in the four regression models were determined at baseline (F4). Afamin levels were modelled as a continuous variable (with effect estimates per 10 mg/L increase) or as a categorical variable (with effect estimates per sex‐specific quartile using quartile 1 as reference). The defined upper and lower limits for the afamin quartiles are presented in Appendix S1. Effect estimates are presented as beta coefficients (β) with their corresponding 95% confidence intervals (CI). Trend tests (*p*_trend_) were performed by treating afamin as a continuous variable.

In the prospective analyses, all models were additionally adjusted for the corresponding indices at F4. Apart from that, the same four models with increasing complexity as for the cross‐sectional analyses were built to assess the impact of the covariates in the prospective analyses.

To assess potential effect modifications by sex and glucose tolerance status (normal glucose tolerance (NGT) versus prediabetes/T2D) on the associations between afamin at baseline and fatty liver indices (NAFLD LFS and HSI) at baseline and follow‐up, we performed stratified analyses for two models. *p*‐values for interaction (*p*_interaction_) were obtained by adding a cross‐product term between plasma afamin and the potential effect modifier.

All data were analysed with R software version 3.6.3 (R Development Core Team, R Foundation for Statistical Computing, Vienna, Austria) using packages dplyr/tidyr/DescTools/ggplot2 and *p* < .05 was considered to indicate statistical significance.

## 3 RESULTS

### 3.1 Baseline characteristics of the study population

Table 1 shows the baseline characteristics of the total study population (*n* = 2349) and stratified by sex. Among all study participants, 45.1% were male. Men had larger waist circumferences, higher haemoglobin A_1c_ levels, higher fasting glucose and fasting insulin levels, lower concentrations of total and HDL cholesterol, as well as higher triacylglycerols and IL‐18 levels than women. Compared to women, men differed in their glucose tolerance status (lower proportion of patients with NGT, but higher proportion with prediabetes and T2D), had higher liver enzyme levels (ALT, AST, GGT) and were also more likely to be current smokers. In addition, the rate of alcohol consumption, hypertension and the use of lipid‐lowering drugs were significantly higher in men. NAFLD LFS, FLI, as well as FIB‐4 index were significantly higher in men than in women. Afamin concentrations were on average 72.5 ± 17.1 mg/L in men and 69.5 ± 15.6 mg/L in women (*p* < .001). Men and women were similar with respect to age, BMI, eGFR, hsCRP concentrations, physical activity, the use of NSAIDs and HSI (Table 1).

**TABLE 1:** Baseline characteristics (F4) of the total study population and stratified by sex.

Variable (at KORA F4)	Total sample *n* = 2349 *n* (%) or mean ± SD	Men *n* = 1059; 45.1% *n* (%) or mean ± SD	Women *n* = 1290; 54.9% *n* (%) or mean ± SD	*p*‐Value
Age (years)	55.9 ± 13.5	56.3 ± 13.8	55.6 ± 13.3	.234
BMI (kg/m^2^)	27.6 ± 4.9	27.8 ± 4.2	27.5 ± 5.4	.109
Waist circumference (cm)	93.3 ± 14.0	99.3 ± 12.3	88.3 ± 13.3	**<.001**
HbA_1c_ (mmol/mol)	37.3 ± 6.5	37.7 ± 7.3	36.9 ± 5.8	.**003**
HbA_1c_ (%)	5.6 ± .6	5.6 ± .7	5.5 ± .5	.**002**
Glucose tolerance status^a^				
NGT	1681 (71.6)	721 (68.1)	960 (74.4)	**<.001**
Prediabetes	373 (15.9)	175 (16.5)	198 (15.3)	
T2D	259 (11.0)	145 (13.7)	114 (8.8)	
Fasting glucose (mmol/L)	5.4 ± 1.0	5.6 ± 1.2	5.3 ± .9	**<.001**
Fasting insulin (μU/mL)	11.3 ± 11.7	12.4 ± 15.3	10.4 ± 7.5	**<.001**
eGFR (mL/min per 1.73 m^2^)	87.8 ± 16.9	87.7 ± 17.0	87.8 ± 16.9	.914
eGFR >30 mL/min per 1.73 m^2^	2344 (99.8)	1056 (99.7)	1288 (99.8)	.663
eGFR <30 mL/min per 1.73 m^2^	5 (.2)	3 (.3)	2 (.2)	
Total cholesterol (mmol/L)	5.6 ± 1.0	5.4 ± 1.0	5.6 ± 1.0	**<.001**
HDL cholesterol (mmol/L)^a^	1.4 ± .4	1.3 ± .3	1.6 ± .4	**<.001**
Triacylglycerols (mmol/L)	1.4 ± .9	1.6 ± 1.1	1.2 ± .8	**<.001**
hsCRP (mg/L)^a^	2.5 ± 5.7	2.3 ± 5.6	2.7 ± 5.8	.147
IL‐18 (pg/mL)^a^	320.3 ± 174.8	353.3 ± 154.9	293.1 ± 185.2	**<.001**
ALT (IU/L)	25.0 ± 15.7	29.9 ± 17.4	21.0 ± 12.8	**<.001**
AST (IU/L)	26.2 ± 10.8	28.5 ± 12.2	24.2 ± 9.0	**<.001**
GGT (IU/L)	36.5 ± 35.0	44.7 ± 40.7	29.7 ± 27.6	**<.001**
Smoking (%)^a^				
Never	1055 (44.9)	358 (33.8)	697 (54.1)	**<.001**
Former	903 (38.5)	506 (47.8)	397 (30.8)	
Current	390 (16.6)	195 (18.4)	195 (15.1)	
Alcohol consumption (g/day)	6.7 ± 8.1	10.0 ± 9.4	4.0 ± 5.4	**<.001**
No alcohol consumption (%)	879 (37.4)	291 (27.5)	588 (45.6)	**<.001**
Physically active (%)	1285 (54.7)	579 (54.7)	706 (54.7)	1.000
Hypertension (%)^a^	884 (37.6)	456 (43.1)	428 (33.2)	**<.001**
Use of NSAIDs (%)^a^	73 (3.1)	37 (3.5)	36 (2.8)	.389
Use of lipid‐lowering drugs (%)^a^	298 (12.7)	159 (15.0)	139 (10.8)	.**003**
Afamin (mg/L)	70.8 ± 18.3	72.5 ± 17.1	69.5 ± 15.6	**<.001**
NAFLD liver fat score	−.8 ± 2.4	−.3 ± 2.8	−1.2 ± 1.8	**<.001**
Hepatic steatosis index	36.4 ± 6.1	36.3 ± 5.6	36.5 ± 6.5	.592
Fatty liver index	47.5 ± 30.3	57.6 ± 27.0	39.2 ± 30.3	**<.001**
Fibrosis‐4 index	1.3 ± .7	1.4 ± .7	1.2 ± .6	**<.001**

*Note*: Descriptive statistics are presented as mean ± standard deviation for continuous variables or as counts (percentage) for categorical variables. Significant differences (*p* < 0.05) are highlighted in bold.

Abbreviations: ALT, alanine transaminase; AST, aspartate transaminase; BMI, body mass index; eGFR, estimated glomerular filtration rate; GGT, gamma‐glutamyl‐transferase; HbA_1c_, haemoglobin A_1c_; HDL, high‐density lipoprotein; hsCRP, high‐sensitivity C‐reactive protein; IL, interleukin; NAFLD, non‐alcoholic fatty liver disease; NGT, normal glucose tolerance; NSAID, non‐steroidal anti‐inflammatory drug; SD, standard deviation; T2D, type 2 diabetes.

^a^ The following variables had missing values: BMI (*n* = 3), HDL cholesterol (*n* = 1), hsCRP (*n* = 2), IL‐18 (*n* = 18), smoking (*n* = 1), hypertension (*n* = 3), glucose tolerance status (*n* = 36), lipid‐lowering drugs (*n* = 1) and use of NSAID (*n* = 1).

### 3.2 Cross‐sectional associations between afamin concentrations and fatty liver and fibrosis indices at KORA F4

An increase in afamin concentrations of 10 mg/L was associated with an increase in NAFLD LFS and HSI at all levels of adjustment (all *p* < .001). Categorisation of afamin concentrations into sex‐specific quartiles revealed a concentration‐dependent association of afamin quartiles with the NAFLD LFS as well as HSI (all *p* < .001) (Table 2).

**TABLE 2:** Cross‐sectional associations between afamin and the NAFLD liver fat score, hepatic steatosis index and fibrosis‐4 index at the baseline examination KORA F4.

	Plasma afamin (per 10 mg/L increase)		Sex‐specific quartiles of serum afamin				
			Quartile 1	Quartile 2	Quartile 3	Quartile 4	*p*_trend_
	*β* (95% CI)	*p*	*β* (95% CI)	*β* (95% CI)	*β* (95% CI)	*β* (95% CI)	
NAFLD liver fat score at F4							
Model 1	.70 (.65, .75)	**<.001**	Reference	.47 (.23, .71)	1.10 (.86, 1.34)	2.69 (2.45, 2.93)	**<.001**
Model 2	.46 (.41, .52)	**<.001**	Reference	.17 (−.05, .39)	.44 (.21, .67)	1.64 (1.39, 1.88)	**<.001**
Model 3	.32 (.27, .37)	**<.001**	Reference	.11 (−.09, .32)	.25 (.04, .47)	1.08 (.84, 1.32)	**<.001**
Model 4	.33 (.28, .38)	**<.001**	Reference	.12 (−.09, .33)	.26 (.04, .48)	1.10 (.86, 1.34)	**<.001**
Hepatic steatosis index at F4							
Model 1	1.77 (1.63, 1.90)	**<.001**	Reference	2.27 (1.64, 2.89)	4.54 (3.91, 5.16)	7.66 (7.04, 8.29)	**<.001**
Model 2	1.33 (1.19, 1.48)	**<.001**	Reference	1.98 (1.39, 2.57)	3.48 (2.88, 4.09)	5.79 (5.15, 6.44)	**<.001**
Model 3	.33 (.26, .39)	**<.001**	Reference	.44 (.18, .70)	.75 (.48, 1.02)	1.38 (1.08, 1.68)	**<.001**
Model 4	.33 (.26, .40)	**<.001**	Reference	.45 (.19, .71)	.74 (.47, 1.01)	1.38 (1.08, 1.68)	**<.001**
Fibrosis‐4 index at F4							
Model 1	.06 (.04, .08)	**<.001**	Reference	.00 (−.08, .09)	.10 (.01, .18)	.22 (.13, .30)	**<.001**
Model 2	.01 (−.01, .03)	.191	Reference	−.03 (−.11, .05)	.02 (−.06, .10)	.04 (−.05, .13)	.218
Model 3	.01 (−.02, .02)	.778	Reference	−.09 (−.17, −.02)	−.04 (−.12, .03)	−.03 (−.11, .06)	.912
Model 4	.00 (−.02, .02)	.851	Reference	−.10 (−.18, −.03)	−.05 (−.13, .03)	−.05 (−.13, .03)	.602

*Note*: Beta coefficients (*β*) with their corresponding 95% confidence intervals (CI) and *p*‐values were calculated using multivariable linear regression analysis. Significant differences (*p* < .05) are highlighted in bold. For the NAFLD liver fat score the following four models with adjustments for covariates were built: Model 1: crude. Model 2: adjusted for age, sex, smoking status, alcohol consumption, physical activity, body mass index, total cholesterol, estimated glomerular filtration rate, lipid‐lowering medication and regular use of non‐steroidal anti‐inflammatory drugs. Model 3: adjusted for model 2 except for total cholesterol, but additionally adjusted for hypertension, triglycerides, total cholesterol to high‐density lipoprotein cholesterol ratio and diabetes. Model 4: adjusted for model 3 and high‐sensitivity C‐reactive protein and interleukin‐18. For the hepatic steatosis index the following four models with adjustments for covariates were built: Model 1: crude. Model 2: adjusted for age, smoking status, alcohol consumption, physical activity, hypertension, triglycerides, total cholesterol to high‐density lipoprotein cholesterol ratio, estimated glomerular filtration rate, lipid‐lowering medication and regular use of non‐steroidal anti‐inflammatory drugs. Model 3: adjusted for model 2 and sex, body mass index and diabetes. Model 4: adjusted for model 3 and high‐sensitivity C‐reactive protein and interleukin‐18. For the fibrosis‐4 index the following four models with adjustments for covariates were built: Model 1: crude. Model 2: adjusted for sex, smoking status, alcohol consumption, physical activity, hypertension status, diabetes mellitus status, body mass index, triglycerides, total cholesterol to high‐density lipoprotein cholesterol ratio, estimated glomerular filtration rate, regular use of non‐steroidal anti‐inflammatory drugs and lipid‐lowering medication. Model 3: adjusted for model 2 and age. Model 4: adjusted for model 3 and for high‐sensitivity C‐reactive protein and interleukin‐18.

Abbreviations: β, beta coefficient; CI, confidence interval; NAFLD, non‐alcoholic fatty liver disease.

In addition, an increment in afamin concentrations of 10 mg/L was positively associated with the FLI at all levels of adjustment (all *p* < .001). When using sex‐specific afamin quartiles, we observed dose‐dependent associations with the FLI (all *p*_trend_ <.001) (Table S1).

Moreover, the cross‐sectional associations between afamin concentrations and the FIB‐4 index as a fibrosis marker were assessed. Higher afamin levels were associated with a higher FIB‐4 index in the unadjusted model in the analysis with afamin as a continuous variable and based on afamin quartiles (*p* and *p*_trend_ < .001). However, these associations disappeared after adjustment for confounders (all *p* and *p*_trend_ >.05) (Table 2).

### 3.3 Prospective associations between afamin concentrations at KORA F4 and fatty liver and fibrosis indices at KORA FF4

Higher afamin levels at KORA F4 (baseline) were associated with higher increases in NAFLD LFS between KORA F4 and FF4 at all levels of adjustment (all *p* < .001), whereas there were no significant associations with HSI in any model (all *p* > .05). When afamin concentrations were categorized in sex‐specific quartiles, results were confirmed for NAFLD LFS. In contrast, afamin levels in quartiles 2–4 were associated with higher increases in HSI compared to quartile 1 but without a linear dose–response relationship (all *p*_trend_ > .05) (Table 3).

**TABLE 3:** Prospective associations of afamin levels at KORA F4 with the NAFLD liver fat score, hepatic steatosis index and fibrosis‐4 index at KORA FF4.

	Plasma afamin (per 10 mg/L increase)		Sex‐specific quartiles of serum afamin				
			Quartile 1	Quartile 2	Quartile 3	Quartile 4	*p*_trend_
	*β* (95% CI)	*p*	*β* (95% CI)	*β* (95% CI)	*β* (95% CI)	*β* (95% CI)	
NAFLD liver fat score at FF4							
Model 1	.39 (.33, .44)	**<.001**	Reference	.40 (.18, .62)	.73 (.51, .96)	1.43 (1.19, 1.67)	**<.001**
Model 2	.28 (.22, .33)	**<.001**	Reference	.24 (.03, .45)	.44 (.21, .66)	.98 (.74, 1.23)	**<.001**
Model 3	.23 (.17, .28)	**<.001**	Reference	.21 (.00, .41)	.33 (.12, .55)	.79 (.54, 1.03)	**<.001**
Model 4	.22 (.17, .28)	**<.001**	Reference	.20 (−.01, .41)	.32 (.10, .53)	.76 (.52, 1.01)	**<.001**
Hepatic steatosis index at FF4							
Model 1	−.04 (−.17, .09)	.574	Reference	1.07 (.54, 1.60)	.51 (−.03, 1.05)	.27 (−.32, .86)	.838
Model 2	.06 (−.08, .19)	.421	Reference	1.14 (.62, 1.67)	.71 (.16, 1.25)	.67 (.06, 1.29)	.306
Model 3	.12 (−.02, .25)	.096	Reference	1.11 (.59, 1.63)	.62 (.09, 1.16)	.76 (.15, 1.36)	.082
Model 4	.12 (−.02, .25)	.097	Reference	1.12 (.60, 1.64)	.62 (.08, 1.16)	.78 (.16, 1.39)	.080
Fibrosis‐4 index at FF4							
Model 1	.02 (.00, .03)	.**018**	Reference	−.01 (−.07, .05)	.00 (−.06, .06)	.05 (−.01, .11)	.**035**
Model 2	.00 (−.01, .02)	.710	Reference	−.03 (−.09, .03)	−.03 (−.09, .04)	.00 (−.07, .07)	.940
Model 3	.00 (−.01, .02)	.904	Reference	−.04 (−.10, .02)	−.04 (−.10, .03)	−.01 (−.08, .06)	.793
Model 4	.00 (−.02, .02)	.973	Reference	−.04 (−.10, .02)	−.04 (−.10, .02)	−.02 (−.09, .05)	.724

*Note*: Beta coefficients (β) with their corresponding 95% confidence intervals (CI) and *p*‐values were calculated using multivariable linear regression analysis. Significant differences (*p* < .05) are highlighted in bold. For the NAFLD liver fat score the following four models with adjustments for covariates were built: Model 1: adjusted for NAFLD liver fat score at F4. Model 2: adjusted for NAFLD liver fat score at F4 and age, sex, smoking status, alcohol consumption, physical activity, body mass index, total cholesterol, estimated glomerular filtration rate, lipid‐lowering medication and regular use of non‐steroidal anti‐inflammatory drugs. Model 3: adjusted for model 3 except for total cholesterol, but additionally adjusted for hypertension, triglycerides, total cholesterol to high‐density lipoprotein cholesterol ratio and diabetes. Model 4: adjusted for model 3 and C‐reactive protein and interleukin‐18. For the hepatic steatosis index the following four models with adjustments for covariates were built: Model 1: adjusted for hepatic steatosis index at F4. Model 2: adjusted for hepatic steatosis index at F4 and age, smoking status, alcohol consumption, physical activity, hypertension, triglycerides, total cholesterol to high‐density lipoprotein cholesterol ratio, estimated glomerular filtration rate, lipid‐lowering medication and regular use of non‐steroidal anti‐inflammatory drugs. Model 3: adjusted for model 2 and sex, body mass index and diabetes. Model 4: adjusted for model 3 and high‐sensitivity C‐reactive protein and interleukin‐18. For the fibrosis‐4 index the following four models with adjustments for covariates were built: Model 1: adjusted for fibrosis‐4 index at F4. Model 2: adjusted for Fibrosis‐4 index at F4 and sex, smoking status, alcohol consumption, physical activity, hypertension, diabetes, body mass index, triglycerides, total cholesterol to high‐density lipoprotein cholesterol ratio, estimated glomerular filtration rate, regular use of non‐steroidal anti‐inflammatory drugs and lipid‐lowering medication. Model 3: adjusted for model 2 and age. Model 4: adjusted for model 3 and high‐sensitivity C‐reactive protein and interleukin‐18.

Abbreviations: β, beta coefficient; CI, confidence interval; NAFLD, non‐alcoholic fatty liver disease.

Afamin levels in quartile 2 were associated with increases in FLI compared to quartile 1. However, prospective analyses did not show any significant associations between plasma afamin and FLI in any model using afamin as a continuous or categorical variable (all *p* and *p*_trend_ > .05) (Table S2).

Finally, in the unadjusted model, higher afamin concentrations were associated with a higher FIB‐4 index (*p* and *p*_trend_ < .05). However, these significant associations disappeared after adjustment for confounders (all *p* and *p*_trend_ > .05) (Table 3).

### 3.4 Effect modifications by sex and glucose tolerance status

To examine potential effect modifications, we tested for interactions by sex and by glucose tolerance status (NGT versus prediabetes/T2D) for models 2 and 3 and performed respective subgroup analyses since interactions were observed. In the cross‐sectional analyses, higher plasma afamin concentrations were associated with more pronounced increases in the NAFLD LFS in men and in people with prediabetes or diabetes (all *p*_interaction_ < .001). The prospective associations between afamin and NAFLD LFS were also stronger in men than in women (all *p*_interaction_ < .05).

In model 2, the cross‐sectional associations between afamin levels and HSI were also more pronounced in men, but lower in people with prediabetes or diabetes compared to the NGT subgroup (both *p*_interaction_ < .001). However, after additional adjustments, the statistical significance was lost (both *p*_interaction_ > .05). In contrast, the prospective associations between afamin and HSI did not differ by sex (both *p*_interaction_ > .05). Finally, higher afamin levels were associated with more pronounced increases in the HSI in the NGT subgroup than in people with prediabetes or diabetes in the prospective analyses, but these associations were only statistically significant in model 3 (*p*_interaction_ = .013) (Table 4).

**TABLE 4:** Effect modifications of sex and glucose tolerance status on the associations of afamin levels with NAFLD liver fat score and hepatic steatosis index at KORA F4 and KORA FF4.

	Sex			Glucose metabolism status		
	Men	Women	*p*_interaction_	NGT	Prediabetes and T2D	*p*_interaction_
	*β* (95% CI)	*β* (95% CI)		*β* (95% CI)	*β* (95% CI)	
NAFLD liver fat score at F4						
Model 2						
*N* (%)	1056 (45.1)	1288 (54.9)	/	1679 (71.6)	629 (26.8)	/
Per 10 mg/L	.56 (.46, .66)	.36 (.30, .41)	**<.001**	.27 (.24, .31)	.64 (.49, .78)	**<.001**
Model 3						
*N* (%)	1037 (45.0)	1268 (55.0)	/	1676 (72.7)	629 (27.3)	/
Per 10 mg/L	.41 (.32, .51)	.22 (.17, .26)	**<.001**	.21 (.17, .24)	.58 (.42, .73)	**<.001**
NAFLD liver fat score at FF4						
Model 2						
*N* (%)	624 (44.2)	787 (55.8)	/	1081 (76.6)	312 (22.1)	/
Per 10 mg/L	.36 (.28, .45)	.12 (.05, .19)	.**033**	.12 (.06, .17)	.39 (.25, .53)	**<.001**
Model 3						
*N* (%)	616 (44.3)	776 (55.7)	/	1080 (77.6)	312 (22.4)	/
Per 10 mg/L	.31 (.22, .39)	.10 (.03, .16)	.**022**	.12 (.06, .17)	.33 (.19, .47)	.786
Hepatic steatosis index at F4						
Model 2						
*N* (%)	1066 (45.1)	1298 (54.9)	/	1688 (71.4)	641 (27.1)	/
Per 10 mg/L	1.40 (1.23, 1.58)	1.17 (.96, 1.39)	**<.001**	1.22 (1.05, 1.39)	1.07 (.79, 1.34)	**<.001**
Model 3						
*N* (%)	1048 (45.0)	1281 (55.0)	/	1688 (72.5)	641 (27.5)	/
Per 10 mg/L	.41 (.30, .51)	.23 (.14, .31)	.090	.32 (.24, .40)	.35 (.22, .48)	.916
Hepatic steatosis index at FF4						
Model 2						
*N* (%)	628 (44.1)	797 (55.9)	/	1087 (76.3)	321 (22.5)	/
Per 10 mg/L	.06 (−.15, .27)	.12 (−.06, .31)	.351	.11 (−.06, .28)	−.03 (−.28, .23)	.356
Model 3						
*N* (%)	621 (44.1)	787 (55.9)	/	1087 (77.2)	321 (22.8)	/
Per 10 mg/L	.11 (−.10, .32)	.14 (−.04, .33)	.722	.14 (−.02, .30)	.05 (−.19, .30)	.**013**

*Note*: Descriptive statistics are presented as absolute number *n* (percentage). Beta coefficients (*β*) with their corresponding 95% confidence intervals (CI) and *p*‐values were derived using subgroup analyses and interaction terms. Bold values indicate statistical significance (*p* < .05). For the NAFLD liver fat score at F4 the following two models with adjustments for covariates were built: Model 2: adjusted for age, sex (not in the sex‐stratified analysis), smoking status, alcohol consumption, physical activity, body mass index, total cholesterol, estimated glomerular filtration rate, lipid‐lowering medication and regular use of non‐steroidal anti‐inflammatory drugs. Model 3: adjusted for model 3 except for total cholesterol, but additionally adjusted for hypertension, triglycerides, total cholesterol to high‐density lipoprotein cholesterol ratio and diabetes (not in the analysis stratified by (pre)diabetes status). For the FF4 analysis, all regression models were additionally adjusted for NAFLD liver fat score at F4. For the hepatic steatosis index at F4 the following two models with adjustments for covariates were built: Model 2: adjusted for age, smoking status, alcohol consumption, physical activity, hypertension, triglycerides, total cholesterol to high‐density lipoprotein cholesterol ratio, estimated glomerular filtration rate, lipid‐lowering medication and regular use of non‐steroidal anti‐inflammatory drugs. Model 3: adjusted for model 2 and body mass index and diabetes (not in the analysis stratified by (pre)diabetes status). For the FF4 analysis, all regression models were additionally adjusted for hepatic steatosis index at F4. β, beta coefficient; CI, confidence interval; NAFLD, non‐alcoholic fatty liver disease; NGT, normal glucose tolerance; T2D, type 2 diabetes.

## 4 DISCUSSION

This study found that higher plasma afamin concentrations were positively associated with the fatty liver indices NAFLD LFS, HSI and FLI in the cross‐sectional analysis. The prospective analysis corroborated these associations between afamin levels and NAFLD LFS. The cross‐sectional associations between afamin and NAFLD LFS were more pronounced in men and in people with prediabetes or diabetes. In the prospective analysis, higher afamin levels were also associated with more pronounced increases in the NAFLD LFS in men.

### 4.1 Cross‐sectional associations between afamin, fatty liver and fibrosis indices

This study found positive cross‐sectional associations between plasma afamin levels and the three fatty liver indices NAFLD LFS, HSI and FLI. This is in line with two previous epidemiological studies demonstrating that afamin concentrations were positively associated with prevalent MASLD.^14^, ^15^ In these studies, MASLD had mostly been diagnosed using abdominal ultrasound examinations, which have potential limitations. Ultrasound provides rather semi‐quantitative estimates, as it was indicated that the sensitivity for the diagnosis of mild steatosis is low.^16^ In contrast, our study is the first that uses three different non‐invasive fatty liver indices that are mainly based on routine laboratory and anthropometric measurements.

Of note, our cross‐sectional analyses showed strong initial effect estimates, but these associations were attenuated differentially with extensive adjustment for confounders. In the extended model 4, we cannot exclude the possibility of over‐adjustment. However, even after adjustment for a large number of potential confounders, the positive associations between afamin and all three fatty liver indices remained significant. Despite all this, we cannot rule out that there are additional factors that were not measured and therefore not tested as potential confounders. In this context, it should be noted that significant inverse associations were reported between afamin and interleukin‐6 (IL‐6) as well as afamin and adiponectin.^27^, ^28^ Therefore, these results should encourage further studies considering additional inflammatory biomarkers, including IL‐6 and the peptide hormone adiponectin, as possible afamin‐regulating factors.

In accordance with our observations, there is only one small observational study with a cross‐sectional design indicating that afamin may be positively correlated with the FLI.^29^ Since the FLI was originally used to assess hepatic steatosis in the general population,^16^, ^25^ we additionally included the non‐alcoholic fatty liver indices NAFLD LFS and HSI in our analyses.^23^, ^24^ Moreover, the use of three fatty liver indices allows for a more robust conclusion since the diagnostic efficacy of the respective indices can be influenced by various factors such as grade of steatosis, stage of health, ethnicity or demographic characteristics and anthropometric factors.^16^, ^30^

Overall, our data add to the current literature. However, our findings should be validated in future studies in which both afamin measurements and MASLD diagnosis using gold‐standard methods such as magnetic resonance imaging (MRI) or spectroscopy (MRS) are available.

To our knowledge, this is also the first study that investigated the association of plasma afamin and FIB‐4 index as indicators of liver fibrosis, although our data did not show a significant link between afamin levels and the FIB‐4 index. The potential reason for this lack of significant associations could be related to the selected study cohort and their characteristics. Considering the cut‐off values of the FIB‐4 index, advanced fibrosis was not present in our study population or study participants were only at low risk of developing advanced fibrosis. In low‐prevalence populations, NITs should be selected to rule out rather than to diagnose the presence of advanced liver fibrosis.^31^ Therefore, associations between afamin and FIB‐4 index should also be tested in populations with more advanced SLD.

Nevertheless, among the NITs, FIB‐4 index and NAFLD fibrosis score offer the best diagnostic performance for detecting advanced fibrosis.^32^ Taken together, we cannot rule out associations between afamin and the FIB‐4 index. Therefore, further epidemiological studies in more suitable cohorts are warranted to clarify this issue.

### 4.2 Prospective associations between afamin, fatty liver and fibrosis indices

The second main finding of this study is the prospective association between higher plasma afamin levels and more pronounced increases in the fatty liver index NAFLD LFS. Our data are novel because our study assessed these associations in older individuals aged 31–82 years in a prospective design. One previous study reported that afamin levels were positively associated with the incidence of MASLD^14^ in younger individuals. Therefore, these findings cannot be generalised to older populations, although these older individuals represent the more relevant age group for MASLD development.^33^

In addition, it is important that a temporal relationship cannot be determined from our cross‐sectional analysis between afamin levels and fatty liver indices. In contrast, the prospective analyses found a temporal relationship that is a prerequisite for a potential causal effect of afamin on MASLD development.

Finally, the strong initial effect estimates were attenuated after comprehensive adjustment for potential confounders, and effect estimates from the extended model 4 are likely over‐adjusted. However, the positive associations between afamin and NAFLD LFS remained statistically significant in all models. Again, potential residual confounding cannot be excluded. Further studies assessing the incremental predictive value of afamin for the development of MASLD over and above established clinical risk factors of MASLD are required.

### 4.3 Effect modifications by sex and glucose tolerance status

No previous study examined the possible effect‐modifying role of sex and glucose tolerance status in the associations between afamin and fatty liver indices, although several population‐based studies showed that the prevalence of MASLD, detected by ultrasonography or the validated FLI, is higher in men than in women^17^, ^18^ and is also more pronounced in people with prediabetes as well as T2D.^19^, ^20^ Here, we present evidence that sex and glucose tolerance status partly act as effect modifiers. We found that cross‐sectional and prospective associations between afamin concentrations and NAFLD LFS were more pronounced in men.

One possible explanation for these observed differences could be the comparatively lower oestrogen levels in men, as one study has suggested that oestrogen protects against MASLD in premenopausal women.^17^ Another explanation may be related to the described differences in triglyceride synthesis, fatty acid oxidation and oxidative stress in men and women.^17^, ^34^

In addition, we found that men had higher plasma afamin levels than women, which points to partly sex‐specific effects on the regulation of afamin expression and release.

Moreover, our study found that cross‐sectional analyses between afamin and NAFLD LFS were higher in individuals with prediabetes or T2D compared to those with NGT. There is convincing evidence that MASLD and T2D have a bidirectional relationship and that both prediabetes and T2D are associated with an increased risk of MASLD.^35^, ^36^, ^37^, ^38^ Of note, significant associations have already been reported between the plasma concentrations of afamin and the prevalence and incidence of T2D.^12^ In line, epidemiological studies reported that afamin positively correlated with parameters of IR, including fasting insulin,^29^, ^39^ HOMA‐IR^12^, ^29^, ^39^, ^40^ and elevated fasting glucose,^12^, ^13^ and negatively with whole‐body insulin sensitivity.^29^ Moreover, NAFLD LFS, HSI and FLI have been proposed as predictors of IR because these indices strongly and inversely correlated with measures of insulin sensitivity.^16^ Collectively, our data show that the associations between afamin and NAFLD LFS differ by sex and glucose tolerance status. Our novel study extends the current literature and should motivate similar analyses in other MRI‐based studies to validate our findings and search for underlying mechanisms.

### 4.4 Pathophysiological link between afamin and MASLD

Possible mechanistic explanations for the association between plasma afamin levels and increases in fatty liver indices could be explained by (i) the interference of afamin in glucose and lipid metabolism, (ii) the induction of oxidative stress by afamin, (iii) the involvement of afamin in insulin resistance or (iv) the contribution of afamin to inflammatory responses.

The first explanation is based on an in vivo study demonstrating that the overexpression of afamin increased the concentrations of total cholesterol, triglycerides and glucose in mice.^13^ With respect to the second explanation, two aspects should be noted. First, afamin has been described as a glycoprotein with vitamin E‐binding properties,^11^ whereby vitamin E is a potent antioxidant. Vitamin E is effective in attenuating the progression of MASLD^41^ through the reduction of oxidative stress in mouse models^42^, ^43^ and the decrease in serum ALT and AST concentrations among MASLD patients.^44^ Second, a significant correlation between serum afamin concentrations and oxidized low‐density lipoprotein levels was found in obese non‐diabetic patients.^45^ Based on these data, afamin‐induced oxidative stress, for example by potential functional inhibition of vitamin E, may represent a mechanism explaining the inverse association of afamin with MASLD development. The third explanation considers the fact that afamin is strongly related to IR^12^, ^29^, ^39^, ^40^ and is positively associated with prevalent and incident MetS and all its individual components.^13^ Therefore, IR could partly mediate the association between afamin and MASLD. The fourth explanation assumes that afamin promotes the wingless‐type MMTV integration site family (Wnt) signalling pathway, which in turn triggers a pro‐inflammatory response, lipogenic outcomes and IR in mature adipocytes. This in turn might make the liver susceptible to the occurrence of MASLD.^46^ In line, it has been shown that Wnt5a induces C‐Jun N‐terminal kinase activation,^47^ which interferes with insulin signalling in the liver^47^, ^48^ suggesting that the non‐canonical Wnt pathway may contribute to hepatic steatosis, MASLD and MASH.^47^

However, further functional studies in hepatic cell models have to be performed to elucidate the exact underlying pathophysiological mechanism(s) explaining our associations between afamin and fatty liver indices and to clarify whether afamin directly contributes to the MASLD process.

### 4.5 Strengths and limitations

A main strength of the current study is that the data was derived from both cross‐sectional and prospective analyses from a large population‐based cohort. Further strengths include the extended follow‐up, the wide age range of study participants, the comprehensive adjustments for potential confounders and the use of three fatty liver indices to assess the relevance of afamin in the pathogenesis of MASLD.

This study has also some limitations. The lack of MRI or liver biopsy data in the F4 examination has to be considered as a main limitation, but these were not possible in this large epidemiological study. Thus, we only studied quantitative phenotypes and could not validate the diagnostic performance of liver indices to identify hepatic steatosis. However, although fatty liver indices could not substitute for fat quantification determined by MRS, the used indices offer modest efficacy to detect hepatic steatosis and might serve as surrogate parameters for liver fat content.^16^ Moreover, we used several of these fatty liver indices which in turn have been selected for ease of application in this large‐scale population study. In addition, we cannot rule out an over‐adjustment due to the comprehensive adjustments for confounders. It is possible that there are further confounders interacting with afamin that were not measured and therefore could not be considered. Finally, the study population included mainly people of European descent so the findings may not be generalisable to other ethnicities.

## 5 CONCLUSIONS

In conclusion, higher afamin concentrations were positively associated with NAFLD LFS in both cross‐sectional and prospective analyses with potential effect modification by sex and glucose tolerance status. These associations should be validated to find out whether afamin has the potential to be a novel biomarker for the non‐invasive diagnosis of MASLD independently of known MASLD risk factors. Future studies need to elucidate whether afamin also contributes to the pathogenesis of MASLD and whether afamin plays a role in hepatic fibrosis.

## AUTHOR CONTRIBUTIONS

CN, HM and CH designed the study. TT, MR, FK and BT contributed to the study design. XC, JN, WR, WK, BK, HD, AP, FK, BT and CH contributed data. FK organized and supervised afamin measurements. CN, AZ, HM and CH drafted the statistical analysis plan. AZ and HM performed the statistical analysis. CN and CH wrote the manuscript. All authors contributed to the discussion of the data, critically revised the manuscript for important intellectual content and approved the final version of the manuscript.

## FUNDING INFORMATION

The KORA study was initiated and financed by the Helmholtz Zentrum München – German Research Center for Environmental Health, which is funded by the German Federal Ministry of Education and Research (BMBF) and by the State of Bavaria. Data collection in the KORA study is done in cooperation with the University Hospital of Augsburg. The German Diabetes Center is supported by the Ministry of Culture and Science of the State of North Rhine‐Westphalia (Düsseldorf, Germany) and the German Federal Ministry of Health (Berlin, Germany). This study was supported in part by a grant from the German Federal Ministry of Education and Research (BMBF) to the German Center for Diabetes Research (DZD) and in part by a grant from the German Diabetes Association (DDG). The funders of the study had no role in study design, data collection, analysis, interpretation, or writing of the manuscript.

## CONFLICT OF INTEREST STATEMENT

The authors declare that there is no conflict of interest regarding this study.

## GUARANTOR STATEMENT

All authors approved the final version of the manuscript. CH is the guarantor of this work and, as such, had full access to all the data in the study and takes responsibility for the integrity of the data and the accuracy of the data analysis.

## Supporting information


Appendix S1.

## Data Availability

The data are subject to national data protection laws. Therefore, data cannot be made freely available in a public repository. However, data can be requested through an individual project agreement with KORA. To obtain permission to use KORA data under the terms of a project agreement, please use the digital tool KORA.PASST (https://epi.helmholtz‐muenchen.de/).
