# Supplementary material for: Higher afamin concentrations are associated with higher fatty liver indices: Population‐based KORA F4/FF4 study
Source: Eur J Clin Invest. 2025 Jul 9;55(12):e70095. doi: 10.1111/eci.70095 (PMC12621296; doi:10.1111/eci.70095)
Supplement: Supplementary file 1 — Appendix S1. [file ECI-55-e70095-s001.pdf]

Higher afamin concentrations are associated with increases in fatty liver indices: population-based KORA F4/FF4 study

Corinna Niersmann, Anna Zhu, Haifa Maalmi, Xinting Cai, Jana Nano, Wolfgang Rathmann, Wolfgang Koenig, Toshinari Takamura, Barbara Kollerits, Hans Dieplinger, Annette Peters, Michael Roden, Florian Kronenberg, Barbara Thorand, Christian Herder

Table of contents

Supplementary methods..…………………………………………………………...pp. 2-4

Supplementary table 1…………………………………………………………..p. 5

Supplementary table 2…………………………………………………………..p. 6

Supplementary figure 1…………………………………………………………p. 7

Supplementary figure 2...p. 8

Supplementary methods

Assessment of covariates:

Briefly, BMI was calculated as body weight (in kilograms) divided by [height (in m)]^2. Waist circumference was measured midway between the lower rib margin and the iliac crest.[1] Haemoglobin A_{1c} was measured using cation-exchange high-performance liquid chromatography photometric assays on an Adams HA 8160 Hemoglobin Analysis System (Arkray Inc., distributed by A. Menarini Diagnostics, Florence, Italy), and total cholesterol, high-density lipoprotein and triacylglycerols were determined using an enzymatic colorimetric CHOL Flex, ALDL Flex or TGL Flex assay on a Dimension RxL instrument (Dade Behring Inc., Newark, USA), respectively.[2] The assessment of the glucose tolerance status was performed using standardised oral glucose tolerance tests as described in detail elsewhere.[3] The following three glucose tolerance categories were defined according to the 1999 World Health Organisation diagnostic criteria[4]: normal glucose tolerance (NGT), prediabetes, and type 2 diabetes (T2D). Prediabetes was defined as impaired fasting glucose (IFG), impaired glucose tolerance (IGT) or combined IFG/IGT.

Kidney function was assessed from the estimated glomerular filtration rate using the Chronic Kidney Disease Epidemiology Collaboration (CKD-EPI) serum creatinine equation.[5]

Information on sex, age, smoking status, alcohol consumption and physical activity were obtained by standardised interviews. Participants were asked whether they were current smokers, past smokers or never smokers.[1] With respect to alcohol consumption, study participants were divided into individuals with or without alcohol intake. Alcohol consumers (>30 g/day for men and >20 g/day for women) were excluded from the analyses. Physical activity was defined as more than 1 h of activity per week during leisure time in summer and in winter.[6] Hypertension was defined by systolic blood pressure more than 140 mmHg or diastolic blood pressure more than 90 mmHg (with or without the use of anti-hypertensive medications). Using a database-supported computer software data on the regular use of non-steroidal anti-

inflammatory drugs (NSAIDs) and lipid-lowering drugs were collected.[1] The liver enzymes ALT, AST and GGT were analysed according to the recommendations of the International Federation of Clinical Chemistry from 1983 (confirmed and extended in 2002) as described.[7] Plasma concentrations of high-sensitivity C-reactive protein were measured using a high-sensitivity latex-enhanced nephelometric assay on a BN II analyzer (Dade Behring) and plasma interleukin-18 levels were determined using the ELISA kits from MBL (Nagoya, Japan) as described before.[8]

References

1. Herder C, Schneider A, Zhang S, Wolf K, Maalmi H, Huth C, Pickford R, Laxy M, Bönhof GJ, Koenig W, Rathmann W, Roden M, Peters A, Thorand B, Ziegler D. Association of Long-Term Air Pollution with Prevalence and Incidence of Distal Sensorimotor Polyneuropathy: KORA F4/FF4 Study. *Environ Health Perspect.* 2020; 128(12):127013. (doi: 10.1289/EHP7311)

2. Laxy M, Knoll G, Schunk M, Meisinger C, Huth C, Holle R. Quality of Diabetes Care in Germany Improved from 2000 to 2007 to 2014, but Improvements Diminished since 2007. Evidence from the Population-Based KORA Studies. *PLoS One.* 2016; 11(10):e0164704. (doi: 10.1371/journal.pone.0164704)

3. Rathmann W, Strassburger K, Heier M, Holle R, Thorand B, Giani G, Meisinger C. Incidence of Type 2 diabetes in the elderly German population and the effect of clinical and lifestyle risk factors: KORA S4/F4 cohort study. *Diabet Med.* 2009; 26(12):1212-9. (doi: 10.1111/j.1464-5491.2009.02863.x)

4. World Health Organization. Definition, Diagnosis and Classification of Diabetes Mellitus and its Complications. Report of a WHO Consultation. Part 1: Diagnosis and Classification of Diabetes Mellitus. Geneva: World Health Organizaion, 1999.

5. Levey AS, Stevens LA, Schmid CH, Zhang YL, Castro AF 3rd, Feldman HI, Kusek JW, Eggers P, Van Lente F, Greene T, Coresh J; CKD-EPI (Chronic Kidney Disease Epidemiology Collaboration). A new equation to estimate glomerular filtration rate. *Ann Intern Med*. 2009; 150(9):604-12. (doi: 10.7326/0003-4819-150-9-200905050-00006) Erratum in: *Ann Intern Med.* 2011; 155(6):408.

6. Herder C, Kannenberg JM, Carstensen-Kirberg M, Huth C, Meisinger C, Koenig W, Peters A, Rathmann W, Roden M, Thorand B. Serum levels of interleukin-22, cardiometabolic risk factors and incident type 2 diabetes: KORA F4/FF4 study. *Cardiovasc Diabetol.* 2017; 16(1):17. (doi: 10.1186/s12933-017-0498-6)

7. Rückert IM, Heier M, Rathmann W, Baumeister SE, Döring A, Meisinger C. Association between markers of fatty liver disease and impaired glucose regulation in men and women from

the general population: the KORA-F4-study. *PLoS One.* 2011; 6(8):e22932. (doi: 10.1371/journal.pone.0022932)

8. Herder C, Bongaerts BW, Rathmann W, Heier M, Kowall B, Koenig W, Thorand B, Roden M, Meisinger C, Ziegler D. Association of subclinical inflammation with polyneuropathy in the older population: KORA F4 study. *Diabetes Care*. 2013; 36(11):3663-70. (doi: 10.2337/dc13-0382)

The following cut-off values were defined for the afamin quartiles (Q) (mg/L) at F4:

NAFLD liver fat score:
Q1 (male: 46.19-60.27; female: 46.19-57.65),
Q2 (male: 60.28-70.60; female: 57.66-67.73),
Q3 (male: 70.66-82.15; female: 67.73-79.07),
Q4 (male: 82.15-119.48; female: 79.07-119.48)

Hepatic steatosis index:
Q1 (male: 46.22-60.08; female: 46.22-57.65),
Q2 (male: 60.12-70.45; female: 57.66-67.73),
Q3 (male: 70.51-82.08; female: 67.73-79.06),
Q4 (male: 82.10-119.73; female: 79.07-119.73)

FIB-4 index:
Q1 (male: 27.90-59.91; female: 25.48-57.69),
Q2 (male: 59.91-69.63; female: 57.76-67.46),
Q3 (male: 69.67-81.87; female: 67.50-78.82),
Q4 (male 81.91- 119.56; female: 78.87-119.56)

The following cut-off values were defined for the afamin quartiles (Q) (mg/L) at FF4:

NAFLD liver fat score:
Q1 (male: 46.19-59.83; female: 46.19-56.72),
Q2 (male: 59.88-70.05; female: 56.72-66.65),
Q3 (male: 70.22-81.95; female: 66.67-78.24),
Q4 (male: 82.08-119.48; female: 78.27-119.48)

Hepatic steatosis index:
Q1 (male: 46.22-59.76; female: 46.22-56.81),
Q2 (male: 59.83-70.01; female: 56.86-67.00),
Q3 (male: 70.05-81.95; female: 67.02-78.31),
Q4 (male: 82.08-119.73; female: 78.39-119.73)

FIB-4 index:
Q1 (male: 27.90-59.91; female: 25.48-57.69),
Q2 (male: 59.91-69.63; female: 57.76-67.46),
Q3 (male: 69.67-81.87; female: 67.50-78.82),
Q4 (male 81.91- 119.56; female: 78.87-119.56)

Supplementary tables

Table S1 Cross-sectional associations between afamin and fatty liver index at KORA F4.

	**Plasma afamin (per 10 mg/L increase)**		**Sex-specific quartiles of serum afamin**				
			**Quartile 1**	**Quartile 2**	**Quartile 3**	**Quartile 4**	***p*trend**
	beta (95% CI)	*p*	beta (95% CI)	beta (95% CI)	beta (95% CI)	beta (95% CI)	
**Fatty liver index at F4**							
Model 1	9.91 (9.33, 10.49)	**<0.001**	Ref	12.61 (9.90, 15.32)	23.87 (21.16, 26.58)	40.47 (37.76, 43.18)	**<0.001**
Model 2	5.52 (5.00, 6.03)	**<0.001**	Ref	7.96 (5.83, 10.09)	14.47 (12.29, 16.64)	23.40 (21.08, 25.71)	**<0.001**
Model 3	1.78 (1.47, 2.08)	**<0.001**	Ref	2.76 (1.56, 3.96)	4.27 (3.03, 5.52)	7.83 (6.48, 9.19)	**<0.001**
Model 4	1.73 (1.43, 2.04)	**<0.001**	Ref	2.59 (1.39, 3.79)	4.14 (2.89, 5.38)	7.57 (6.20, 8.94)	**<0.001**

Beta coefficients with their corresponding 95% confidence intervals (CI) and p-values were calculated using multivariable linear regression analysis. Significant differences ($p<0.05$) are highlighted in bold.

The following upper and lower limits were defined for the afamin quartiles (mg/L): Q1 (male: 46.42-59.91; female: 46.42-57.66), Q2 (male: 59.99-69.73; female: 57.67-67.44), Q3 (male: 69.73-81.87; female: 67.44-78.81), Q4 (male: 81.91-119.60; female: 78.82-119.60).

The following four models with adjustments for covariates were built: Model 1: crude. Model 2: adjusted for age, sex, smoking status, alcohol consumption, physical activity, hypertension, total cholesterol to high-density lipoprotein cholesterol ratio, diabetes, glomerular filtration rate, lipid-lowering medication, and regular use of non-steroidal anti-inflammatory drugs. Model 3: adjusted for model 2 and body mass index and triglycerides. Model 4: adjusted for model 3 and high-sensitivity C-reactive protein and interleukin-18.

CI, confidence interval; Q1, quartile 1; Q2, quartile 2; Q3, quartile 3; Q4, quartile 4; Ref, reference.

Table S2 Prospective associations of afamin levels at KORA F4 with fatty liver index at KORA FF4.

	**Plasma afamin (per 10 mg/L increase)**		**Sex-specific quartiles of serum afamin**				
			**Quartile 1**	**Quartile 2**	**Quartile 3**	**Quartile 4**	p_{trend}
	beta (95% CI)	*p*	beta (95% CI)	beta (95% CI)	beta (95% CI)	beta (95% CI)	
**Fatty liver index at FF4**							
Model 1	-0.34 (-0.80, 0.12)	0.142	Ref	2.17 (0.39, 3.94)	0.90 (-0.93, 2.73)	-1.18 (-3.17, 0.81)	0.113
Model 2	-0.12 (-0.59, 0.36)	0.627	Ref	2.51 (0.70, 4.31)	1.42 (-0.45, 3.29)	-0.16 (-2.24, 1.93)	0.476
Model 3	0.05 (-0.42, 0.52)	0.828	Ref	2.49 (0.71, 4.27)	1.55 (-0.30, 3.40)	0.47 (-1.60, 2.53)	0.948
Model 4	0.12 (-0.36, 0.59)	0.633	Ref	2.68 (0.89, 4.47)	1.75 (-0.11, 3.61)	0.74 (-1.35, 2.83)	0.864

Beta coefficients with their corresponding 95% confidence intervals (CI) and p-values were calculated using multivariable linear regression analysis. Significant differences ($p<0.05$) are highlighted in bold.
The following upper and lower limits were defined for the afamin quarters (mg/L): Q1 (male: 46.42-59.76; female: 46.42-56.94), Q2 (male: 59.83-69.75; female: 56.94-66.45), Q3 (male: 69.79-81.58; female: 66.48-77.96), Q4 (male: 81.61-119.60; female: 77.97-119.60).
The following four models with adjustments for covariates were built: Model 1: adjusted for FLI index at F4. Model 2: adjusted for FLI index at F4 and age, sex, smoking status, alcohol consumption, physical activity, hypertension, total cholesterol to high-density lipoprotein cholesterol ratio, diabetes, glomerular filtration rate, lipid-lowering medication, and regular use of non-steroidal anti-inflammatory drugs. Model 3: adjusted for model 2 and body mass index and triglycerides. Model 4: adjusted for model 3 and high-sensitivity C-reactive protein and interleukin-18. CI, confidence interval; Q1, quartile 1; Q2, quartile 2; Q3, quartile 3; Q4, quartile 4; Ref, reference.

Supplementary figures

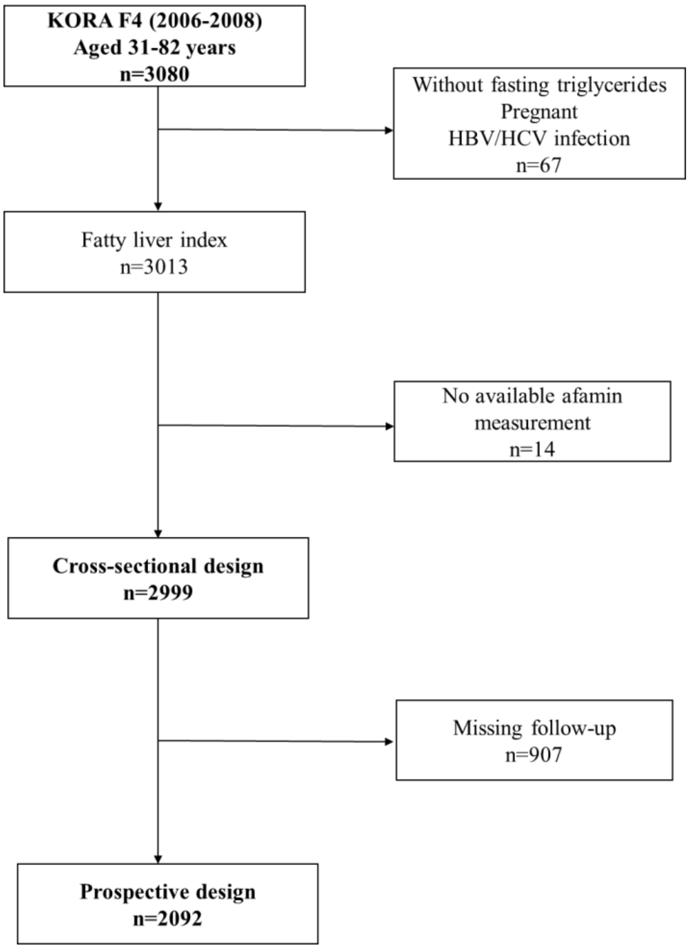

Fig. S1 Flow chart of the inclusion process of the study participants for the analyses of fatty liver index.
HBV, hepatitis B virus; HCV, hepatitis C virus; KORA, Cooperative Health Research in the Region of Augsburg.

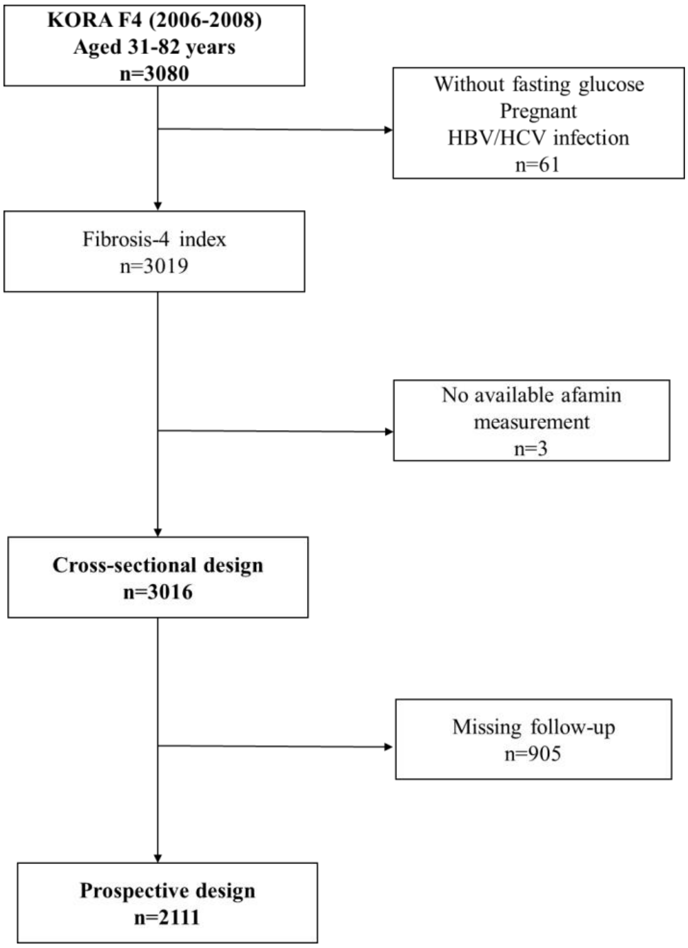

Fig. S2 Flow chart of the inclusion process of the study participants for the analyses of fibrosis-4 index.

HBV, hepatitis B virus; HCV, hepatitis C virus; KORA, Cooperative Health Research in the Region of Augsburg.
